# Decorrelative network architecture for robust electrocardiogram classification

**DOI:** 10.1016/j.patter.2024.101116

**Published:** 2024-12-09

**Authors:** Christopher Wiedeman, Ge Wang

**Affiliations:** 1 Department of Electrical and Computer Systems Engineering, Rensselaer Polytechnic Institute, Troy, NY 12180, USA; 2 Department of Biomedical Engineering, Rensselaer Polytechnic Institute, Troy, NY 12180, USA

**Keywords:** artificial intelligence, AI, deep learning, adversarial defense, electrocardigram, ECG, ensemble learning, uncertainty estimation, robustness

## Abstract

To achieve adequate trust in patient-critical medical tasks, artificial intelligence must be able to recognize instances where they cannot operate confidently. Ensemble methods are deployed to estimate uncertainty, but models in an ensemble often share the same vulnerabilities to adversarial attacks. We propose an ensemble approach based on feature decorrelation and Fourier partitioning for teaching networks diverse features, reducing the chance of perturbation-based fooling. We test our approach against white-box attacks in single- and multi-channel electrocardiogram classification and adapt adversarial training and DVERGE into an ensemble framework for comparison. Our results indicate that the combination of decorrelation and Fourier partitioning maintains performance on unperturbed data while demonstrating superior uncertainty estimation on projected gradient descent and smooth adversarial attacks of various magnitudes. Furthermore, our approach does not require expensive optimization with adversarial samples during training. These methods can be applied to other tasks for more robust models.

## Highlights

• Adversarial attacks can confidently fool ensemble networks in ECG analysis
• Diversifying the extracted features between networks is crucial for robustness
• Feature decorrelation and Fourier partitioning diversify network features

## The bigger picture

The rising demand for medical services has accelerated a healthcare shortage across multiple countries. Artificial intelligence (AI) can potentially help ease this shortage by automating data processing and analysis in clinical workflows. However, current models lack the transparency and robustness required to be trusted with patient-critical tasks. Adversarial attacks are targeted input perturbations that can cause errors in a model’s predictions. These attacks are concerning, having been demonstrated in contexts like electrocardiogram analysis, and could undermine model reliability and facilitate medical fraud. Current methods for resolving this weakness, such as adversarial training, do not scale well to high-dimensional inputs, such as most medical signals.

One alternative is to detect unstable inputs using an ensemble of networks, allowing uncertain cases to be reviewed by human experts. However, adversarial attacks can often fool multiple networks simultaneously, as these models often share the same vulnerabilities. We propose two methods for diversifying these weaknesses among an ensemble. Our electrocardiogram classification experiments show that ensembles trained using these methods are more resilient to adversarial attacks, exhibiting higher uncertainty under unstable inputs while maintaining performance on unperturbed samples.

## Graphical abstract

**Figure:**
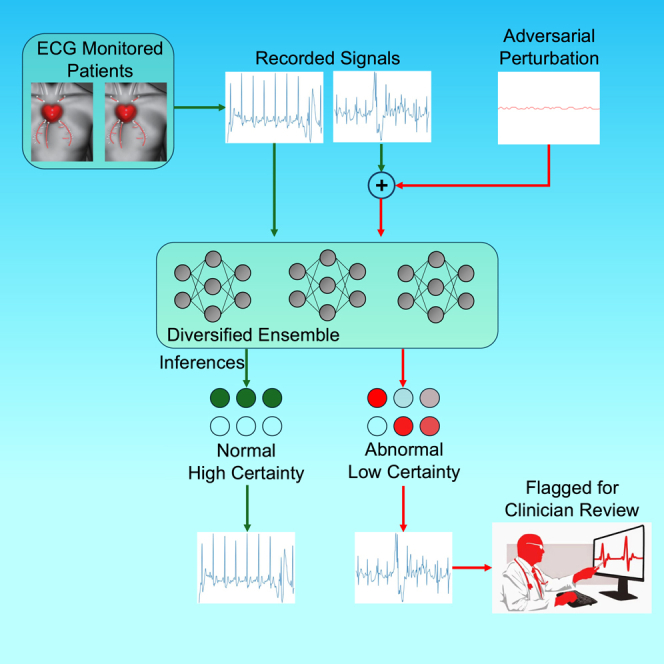

## Teaser

In patient-critical applications like electrocardiogram (ECG) analysis, it is essential for AI to identify instances where it cannot operate effectively. Ensemble methods, which employ multiple networks, can estimate prediction uncertainty but are still deceived by targeted input noise known as adversarial attacks. The authors propose two methods—feature decorrelation and Fourier partitioning—that diversify individual networks, improving robustness against adversarial attacks in ECG analysis. Both methods are scalable and transferable, offering a promising approach to improve robustness across various tasks.

## Introduction

The exponential increase in high-dimensional patient datasets and constant demand for personalized healthcare justify the urgent need for artificial intelligence (AI) in medicine. As an excellent example, electrocardiograms (ECGs) are commonly used for inpatient monitoring of cardiac conditions and are now available in smart or implantable devices. While the proper application of continuous ECG monitoring requires further clinical investigation, large-scale collection and analysis of ECGs, in either inpatient or outpatient populations, has the potential to improve healthcare by monitoring for signs of heart problems or alerting medical services to emergency situations. Deeper analysis of numerous samples is necessary to extract more healthcare-relevant information hidden in these signals. Big data can be leveraged in this instance, but it is infeasible for human clinicians to individually analyze all these recordings, making AI a natural solution to this problem.^1^^,^^2^^,^^3^

For this purpose, many researchers have applied deep learning to ECG classification. The 2017 PhysioNet Challenge is a milestone in this field, where deep neural networks (DNNs) were trained to classify atrial fibrillation (AFib) from single-lead ECGs.^4^ Similarly, the 2018 China Physiological Signal Challenge (CPSC 2018) observed classification of several rhythm abnormalities from 12-lead ECGs.^5^ Top-scoring models can often achieve high classification accuracies on test data, but their interpretability and robustness are major concerns.^6^

Robustness in machine learning is a persistent topic, yet it lacks a unified definition. Broadly, it can be defined as the ability of a model to adapt and perform under changing conditions, but there is no consensus on exactly how these conditions should be defined. For example, a review from Drenkow et al. proposes a unified definition of robustness in computer vision as a model’s performance on images where “environmental, sensor, or rendering conditions are low-probability” (e.g., unusually poor lighting conditions).^7^ Taori et al. focus on computer vision robustness in the context of natural distribution shifts caused by unanticipated changes in the scene.^8^ Another line of work defines robustness with deep belief networks as the ability of the model to accurately approximate a variety of underlying functions or control laws.^9^^,^^10^ This work specifically studies robustness against adversarial attacks, which have been demonstrated both in machine learning broadly and in specific healthcare tasks.

### Adversarial attacks background

Adversarial attacks are small input perturbations that do not change the semantic content yet cause massive errors in a network output, for example, imperceivable noise patterns that, when added to an image, cause a model to misclassify the image.^11^ Given a target model and input, projected gradient descent (PGD) is the most common algorithm for finding adversarial perturbations under an $l_{\infty }$ bound.^12^^,^^13^ Various other algorithms exist, including the use of generative adversarial ensembles.^14^^,^^15^^,^^16^ Impressively, *universal adversarial perturbations* can be crafted to fool a network when added to any sample.^17^^,^^18^

The understanding of adversarial attacks has rapidly developed over the past several years. Akhtar and Mian wrote a broad survey on adversarial attacks in computer vision.^19^ Although adversarial instability may relate to overfitting, DNNs often generalize well to unseen data yet fail on previously seen data that are only slightly altered.^20^ Furthermore, it has been shown that linear models and other machine learning methods are also vulnerable to adversarial attacks.^21^ Early research attributed this phenomenon to a lack of data in high-dimensional problems, which leaves large portions of the total “data manifold” unstable.^22^^,^^23^ The literature has also reported a relationship between large local Lipschitz constants (with regard to the loss function) and adversarial instability.^24^^,^^25^^,^^26^ To our knowledge, the most unifying explanation is the robust features model, where it is shown that data distributions often exhibit statistical patterns that are meaningless to humans but correlate well with different classes.^27^ From a human’s perspective, these patterns are arbitrary and easily perturbed, but since models are trained to maximize only distributional accuracy, they have no reason to prioritize human-favored features over these patterns.

Training models for defending against adversarial attacks remains an open problem, affecting nearly every application of machine learning. Early attempts at defense methods by obfuscating the loss gradient were found to beat only weak attackers, proving ineffective for sophisticated attackers.^28^^,^^29^^,^^30^^,^^31^^,^^32^ Adversarial training, in which a model is iteratively trained on strong adversarial samples, has shown the best results in terms of adversarial robustness.^12^^,^^33^ However, the network size and computational time required are considerable for small problems, and improving adversarial robustness appears to sacrifice performance on clean data.^34^ Satisfactory performance on larger problems has not been achieved due to these limitations.

Another troubling, well-documented characteristic of these attacks is their *transferability*: models trained on the same task will often be fooled by the same attacks, despite having different parameters.^11^^,^^21^^,^^35^ This phenomenon is largely congruent with the robust features model, since these models are likely learning the same useful, but non-robust features. Nevertheless, transferability makes black-box attacks viable, where a malicious attacker does not need detailed knowledge of the target model.

Adversarial instability is particularly alarming for AI deployment in healthcare, where there is ample incentive and opportunity to leverage these attacks for medical fraud, as explained by Finlayson et al..^36^ To summarize, unsavory clinicians and institutions can already engage in “upcoding,” where unnecessary diagnoses are selected to maximize profits.^37^^,^^38^ This malpractice not only increases national healthcare costs, but also burdens patients with inappropriate treatments. To counter this, insurance companies set standards of proof for these decisions and will likely require algorithmic guarantees when AI models are involved. In workflows where deep learning models augment decision-making, malware that intercepts an input before it is analyzed can adversarially perturb the signal, triggering a profitable inference with no change to the model. Detecting such cases of foul play, even with trained readers, is difficult because the ground truth is often ambiguous in medical signal analysis, and the corrupted samples can be indistinguishable from benign samples.

Aside from targeted attacks, the mere existence of adversarial vulnerability breeds a “confidence crisis” in deep models: a model’s adversarial vulnerability reflects its reliance on spurious correlations in a dataset rather than true semantic understanding of the input.^27^ As such, it is difficult to trust these models in patient-critical tasks, where external factors, such as changes in recording conditions, could alter these correlations in unanticipated ways. In other words, there is no guarantee that perturbations along adversarial subspaces would not naturally occur during deployment. Consequently, protection against adversarial attacks is critical for public trust in and responsible deployment of medical AI.

Han et al. have shown that models trained for ECG classification are concerningly susceptible to natural-looking adversarial attacks.^39^ In short, the authors observed that traditional $l_{\infty }$-bounded PGD attacks produce square-wave artifacts that are not physiologically plausible in ECGs; to rectify this, the perturbation space was modified by applying Gaussian smoothing kernels in the attack objective, rendering plausible yet still highly effective adversarial samples.

### Electrocardiography background

The ECG is a front-line, non-invasive tool for monitoring heart health. Skin electrodes measure electrical signals originating from the heart; the behavior of these signals over time corresponds to various events during the cardiac cycle. A healthy rhythm consists of a *P wave, QRS complex*, and *T wave*, which correspond to atrial depolarization, ventricular depolarization, and ventricular repolarization, respectively. Clinical ECGs have traditionally used 12 or 5–7 (Holter) leads, but single-channel ECGs have become more prevalent for continuous monitoring.^40^ These devices are either external or implantable loop recorders (ILRs) designed to record for multiple years.

A variety of downstream analytical tasks are associated with ECG, including biometric identification, respiratory estimation, emotional monitoring, and even fetal heartbeat monitoring. However, the most common application is detection of various arrythmias, which can indicate disorders or disease risk.^40^ AFib, or an abnormally rapid atrial firing rate, is commonly assessed (such as in the 2017 PhysioNet Challenge^4^), but many arrhythmic classes exist, including left or right bundle branch block, premature atrial or ventricular contraction, ventricular fibrillation, tachycardia, and myocardial infarction (heart attack) itself. Certain classes require immediate and serious medical intervention, while others, such as AFib, are not immediately harmful but could still indicate risk of disease. The clinical utility of AFib detection is still an active area of investigation: a systematic review found AFib to be associated with increased risk of myocardial infarction in patients without coronary heart disease and increased risk of all-cause mortality and heart failure in all patients, implying value in detecting it.^41^ On the other hand, a randomized control trial using ILR in patients with at least one risk factor for stroke concluded that continuously monitoring for AFib in this population did not reduce the risk of stroke.^42^ This suggests that detecting AFib early may not provide additional information for managing stroke in patients who are already known to be at risk of the event. Nevertheless, ECG and its subsequent analysis are widely applied and investigated for their implications on patient cardiovascular health.

Clinician review is often required for ECG analysis; this process is resource consuming, especially in the case of continuous monitoring or large patient samples. Thus, automatic classification of these signals is desirable, but simple rule-based classifications often fail to generalize due to data heterogeneity between patients and the non-stationary nature of the signal within patients. As such, researchers have turned to data-driven techniques and machine learning to build ECG classification models. Convolutional neural networks (CNNs) have been the most dominant architecture for ECG arrhythmia classification, but deep belief networks, recurrent neural networks, long short-term memory, and gated recurrent units have all been investigated for the same task.^43^ For an extensive background, Merdjanovska et al. provide a comprehensive review of applications, public datasets, and deep learning research for ECG, and Ebrahimi et al. further survey common deep learning architectures for ECG.^40^^,^^43^

### Uncertainty estimation in healthcare applications

As misdiagnosis in healthcare contexts can cause serious harm, the standard of trust required for AI to operate in this space is high. Rather than replacing clinicians, we envision AI tools augmenting clinical workflows by monitoring inputs over a large population, flagging abnormal or low-confidence instances for human review. To achieve this synergy, models must be capable of gauging their own confidence, recognizing conditions where they can and cannot perform well.^44^ Bayesian deep learning (BDL) is a promising field that models the parameters of a DNN as a distribution rather than a point estimation; sampling this distribution at inference time then allows one to estimate model certainty in an inference.^45^^,^^46^ Approaches for approximating and sampling the parameter distribution, including variational inference and Markov chain Monte Carlo with Hamiltonian dynamics, are often difficult to scale to large spaces.^47^^,^^48^ One simple approach is to train an ensemble of networks for the same task, with each network acting as a sample of the parameter space.^49^ However, this approach does not guarantee robustness: adversarial attacks in particular are known to transfer between different models, because these models (even with vastly different parameters) often learn the same unstable features. Furthermore, in high-dimensional problems with large parameter spaces, training, storing, and running inferences from numerous models quickly become infeasible. As such, the goal in training such ensembles should be to achieve adequate robustness and feature diversity with a small number of models.

### Diversifying features in deep ensembles

To our knowledge, prior work on adversarial robustness has primarily quantified either white-box accuracy or black-box transferability but has not evaluated uncertainty via BDL. Furthermore, works in this field primarily test methods on lower-dimension datasets, such as MNIST or CIFAR10. Our goal is to efficiently train small but diverse deep ensembles capable of gauging uncertainty in worst-case scenarios, i.e., adversarial attacks. We contextualize this in the aforementioned ECG classification, a problem that is much higher in dimension. Adversarial training as a means of diversifying an ensemble is explored, and we also introduce two diversification methods that do not require adversarial sample computations, adding almost no overhead to the regular training process.

According to the robust features model, simply training networks with different parameters in isolation does not achieve adversarial robustness, as networks trained under the same conditions tend to converge toward the same learned features and vulnerabilities.^27^^,^^50^ As such, rather than achieving diversity in the parameter space, we turn the conversation to diversity in the feature space. A mechanism for incentivizing networks to learn different features is necessary. To this end, Yang et al. conceived DVERGE, which diversifies the learned features and adversarial weaknesses in a classification ensemble.^51^ However, this method requires full or partial computations of adversarial samples and round-robin-style training of networks, which necessitate considerable computation capability. In this work, we experiment with ensemble diversification methods that are based on adversarial gradients and other methods that are agnostic to these calculations.

Adversarial training is the best known defense against adversarial attacks and essentially consists of training a network on adversarial samples.^12^ Unfortunately, adversarial training is computationally expensive and reduces accuracy on natural data.^34^

Conventional adversarial training is the best known defense against adversarial attacks, but it does not detect attacks or quantify uncertainty; rather, it attempts to make a single network more robust to attacks. Furthermore, it achieves satisfactory performance only on small problems using large networks to fit more complex decision boundaries.^12^ As such, we adapt *adversarial trained ensembles*, in which individual networks are adversarially trained for additional time after natural training. Consequently, we are able to study adversarial training in the Bayesian ensemble framework and observe how vulnerabilities may be diversified between models.

We propose two distinct methods for diversifying learned features and test these against adversarial ECG attacks in Han et al.^39^ The first method, linear feature decorrelation, is based on previous work,^50^ which not only found a strong linear correlation in the latent space of networks trained on the same task but also found that adding a loss term to reduce the linear correlation greatly decreased the transferability of adversarial attacks. However, the decorrelation process proposed in that work is expensive, as it requires large batch sizes and parallel training of networks. We modify this decorrelation process to make it scalable to larger problems. The second method, which we refer to as Fourier partitioning, is heuristically simpler, employing linear time-invariant filters to partition the input space by frequency, forcing networks to learn features in different frequency bands. This method is inspired by recently discovered connections between the Fourier space and adversarial vulnerability, which demonstrated not only that neural networks can make accurate inferences by relying only on low- or high-frequency characteristics but also that most robustifying training methods only shift a network’s sensitivity to different frequency bands.^34^ As such, we find that a crude but efficient way to teach networks different features is to partition the original inputs by frequency, feeding data in different bands to different networks, and integrate their outputs via ensemble learning.

## Results

### Overview

Two ECG datasets were used for all experiments: the 2017 PhysioNet challenge data (single channel, four classes)^4^ and the 2018 CPSC data (12 channels, nine classes).^5^ The following ensemble training strategies were tested, as well as combinations of methods (e.g., dec + part):

(1) baseline: conventionally trained ensemble, where all models are identically and independently trained
(2) dec: ensemble trained with the proposed linear feature decorrelation to diversify the model features
(3) part: ensemble trained using the proposed Fourier partitioning scheme
(4) adv: a baseline ensemble that undergoes additional ensemble adversarial training
(5) dverge: a baseline ensemble that undergoes additional DVERGE training

Ensembles produce multiple inferences, which can be processed in various ways to gauge epistemic and aleatoric uncertainty.^52^^,^^53^ Here, we adopt a normalized uncertainty approach from Mobiny et al.,^54^ which calculates a normalized measure of certainty $I_{norm}$ based on the mutual information between the sample and the model parameters (see methods, “ensemble training and inference”).

**Figure 1:**
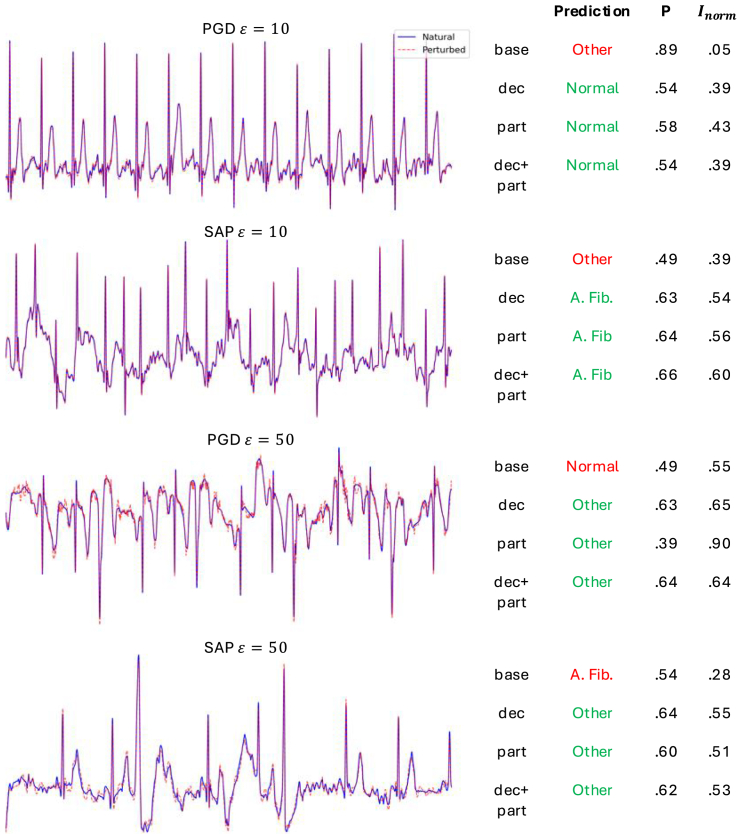
ECG adversarial attack examples Left: examples of projected gradient descent (PGD) and smooth adversarial perturbations (SAP) in the ECG dataset. Right: correct (green) or incorrect (red) aggregate inferences of each ensemble network (normal rhythm, atrial fibrillation, other rhythm, or noise) along with the inferred class probability *P* and normalized uncertainty score $I_{norm}$. Data are from PhysioNet 2017.

We tested each ensemble using validation data perturbed by both PGD and physiologically feasible smooth adversarial perturbations (SAP) attacks of varying magnitude ε, targeting the first model in each ensemble.^39^ Both attack algorithms were adapted to target all models in an ensemble as a white-box attack (see methods, “adversarial attacks,” for details). Figure 1 displays several example attacks from the PhysioNet 2017 data along with inferences, probability, and uncertainty values outputted by the baseline, dec, part, and dec + part ensembles.

Notably, we found that implementing the decorrelation step only slightly increased training time: conventional training took about 352 and 358 min per model on average for PhysioNet and CPSC, respectively; decorrelation added only about 10 min on average to this time in both cases (see methods, “experimental details,” for training parameters). Fourier partitioning did not noticeably increase training time.

### Uncertainty and accuracy performance

To classify an ensemble inference as either certain or uncertain, a threshold, $I_{T}\in \left[0,1\right]$, can be applied to differentiate certain $I_{norm}\leq I_{T}$ and uncertain $I_{norm}>I_{T}$ predictions. A robust model is generally certain when it is correct and uncertain when it is incorrect. Thus, in addition to inference accuracy, we adopt the following three evaluation metrics from Mobiny et al.^54^:

(1) Correct-certain ratio $R_{cc}\left(I_{T}\right)=P_{I_{T}}\left(correct|certain\right)$: probability that the model inference is correct when it is certain
(2) Incorrect-uncertain ratio $R_{iu}\left(I_{T}\right)=P_{I_{T}}\left(uncertain|incorrect\right)$: probability that the model is uncertain when it is incorrect
(3) Uncertainty accuracy $UA\left(I_{T}\right)=P_{I_{T}}\left(correct\cap \;certain\cup \;incorrect\cap \;uncertain\right)$: probability of a desired outcome (either correct and certain or uncertain and incorrect).

All three measures depend on the variable uncertainty threshold $I_{T}$. Thus, similar to a binary classifier, the overall efficacy can be found by integrating the measure as a function of $I_{T}\in \left[0,1\right]$ (i.e., finding the area under the curve [AUC], where larger values are more desirable).

Table 1 summarizes the average prediction accuracy and AUCs for the correct-certain ratio, incorrect-uncertain ratio, and uncertainty accuracy (*UA*) for natural, PGD, and SAP adversarial datasets for the PhysioNet 2017 experiments. Similarly, Table 2 reports the same metrics for the CPSC 2018 experiments. Some initial observations from these numbers are as follows: (1) for the PhysioNet 2017 experiments, dec, part, and dec + part generally have metrics that are comparable or superior to the baseline on natural (ε=0) samples, but dec achieves better performance on stronger ε=50,75,100 PGD and SAP attacks. Although part does not benefit accuracy, the method shows increased *UA*, with dec + part yielding the best *UA* on stronger attacks. adv slightly decreases natural accuracy but improves performance on all adversarial attacks, with the combination of dec + adv generally leading to superior performance in these instances. dverge is comparable to the baseline performance in this instance. (2) On the CPSC 2018 experiments, adv and dec + adv achieve improved robustness on perturbed data but sacrifice considerable accuracy on natural samples. dverge experiences a mild reduction in natural accuracy for a moderate increase on perturbed data, although this advantage diminishes with higher magnitude attacks. The combination of dec + part poses better performance on perturbed data relative to the baseline without sacrificing performance on natural samples.

**Table 1:** Performance metrics on PGD and SAP adversarial samples of varying magnitudes on the PhysioNet 2017 dataset

	0	Attack strength ϵ (PGD)				Attack strength ϵ (SAP)			
		10	50	75	100	10	50	75	100
**Accuracy (%)**									

baseline	83.82	66.82	2.81	1.41	0.82	68.93	3.28	1.41	0.59
dec	84.41	68.23	15.12	6.68	3.52	68.46	12.08	3.63	1.64
part	86.75∗	58.97	2.58	0.35	0.35	63.54	2.93	0.35	0.12
dec + part	85.58	58.97	5.63	1.06	0.59	67.06	3.87	0.82	0.35
adv	83.35	78.55∗	53.34	25.79	9.61	79.48∗	59.20	35.99	17.23
dverge	84.17	61.43	3.05	1.06	0.82	63.42	2.81	1.06	0.70
dec + adv	80.07	76.79	58.62∗	41.74∗	23.68∗	77.37	62.37∗	47.71∗	28.96∗
dec + part + adv	81.48	77.49	53.93	31.42	15.36	78.08	59.44	40.91	21.45

$R_{cc}$**(% AUC)**									

baseline	85.46	69.27	2.30	1.11	0.65	71.41	2.47	1.10	0.42
dec	86.18	71.96	10.98	4.76	2.66	72.53	9.95	2.81	1.10
part	88.18∗	61.96	2.04	0.27	0.33	67.45	2.14	0.25	0.14
dec + part	87.64	67.17	11.22	2.99	2.02	71.90	7.54	1.86	0.84
adv	85.08	80.74∗	57.24	28.28	9.95	81.65∗	63.15∗	39.20	17.99
dverge	86.25	64.59	2.29	0.88	0.76	66.70	2.18	0.90	0.59
dec + adv	81.20	77.98	58.18∗	39.47∗	21.12∗	78.61	62.22	46.19∗	26.33∗
dec + part + adv	83.68	80.15	57.22	32.25	12.91	80.77	61.59	37.44	15.29

$R_{iu}$**(% AUC)**									

baseline	13.77	16.84	15.93	15.09	14.72	17.38	17.05	14.01	11.54
dec	15.91	25.77	43.74	46.92	49.49	25.56	46.33	49.12	49.39
part	15.27	23.27	31.36	30.58	38.70	23.29	26.26	24.65	32.41
dec + part	19.57	42.05∗	68.27∗	69.66∗	67.92∗	31.93∗	66.76∗	68.93∗	65.67∗
adv	16.97	18.30	22.81	27.66	29.04	18.47	22.60	27.32	27.37
dverge	18.59	19.50	19.67	15.68	11.65	20.08	20.17	15.73	9.21
dec + adv	10.58	10.64	10.89	14.69	20.04	10.72	9.88	11.84	13.94
dec + part + adv	21.05∗	22.05	32.04	43.34	55.07	22.09	31.87	44.37	58.06

UA**(% AUC)**									

baseline	81.65	66.39	17.49	15.85	15.17	68.02	18.62	14.79	11.86
dec	81.31	66.80	44.15	46.84	49.34	67.89	46.85	49.06	49.23
part	82.74∗	59.94	32.11	30.68	38.79	64.56	27.23	24.77	32.47
dec + part	81.07	63.47	67.72∗	69.63∗	67.98∗	66.95	66.40∗	68.80∗	65.67∗
adv	78.04	74.68∗	57.19	42.04	33.64	75.48∗	61.04	47.37	36.36
dverge	80.51	62.38	20.98	16.28	12.24	64.02	21.40	16.35	9.69
dec + adv	77.43	74.35	56.33	42.35	32.93	74.97	59.80	46.81	32.83
dec + part + adv	74.40	71.31	55.60	49.25	52.93	72.04	57.03	48.05	52.85

$R_{cc}$, correct-certain ratio; $R_{iu}$, incorrect-uncertain ratio; UA, uncertainty accuracy. Asterisks indicate the best score for a metric.

**Table 2:** Performance metrics on PGD and SAP adversarial samples of varying magnitudes on the CPSC 2018 dataset

	0	Attack strength ϵ (PGD)				Attack strength ϵ (SAP)			
		0.025	0.05	0.15	0.175	0.025	0.05	0.15	0.175
**Accuracy (%)**									

baseline	80.28∗	33.02	13.46	0.00	0.00	29.11	7.51	0.00	0.00
dec	79.66	28.79	13.30	0.00	0.00	24.88	6.89	0.00	0.00
part	79.81	23.00	2.03	0.00	0.00	23.47	1.10	0.00	0.00
dec + part	80.28∗	24.41	8.76	0.00	0.00	24.41	5.63	0.00	0.00
dverge	77.93	38.34	17.68	0.16	0.00	35.37	15.02	0.00	0.00
adv	58.37	40.38	21.75	1.25	0.94	40.38	21.75	1.25	0.94
dec + adv	58.06	45.85	33.96∗	10.80	8.14	45.07	33.18∗	8.92	3.76∗
dec + part + adv	63.85	46.32∗	30.83	13.30∗	8.45∗	46.17∗	30.20	10.17∗	2.66

$R_{cc}$**(% AUC)**									

baseline	84.92	34.60	12.02	0.00	0.00	30.29	5.34	0.00	0.00
dec	83.90	31.91	11.32	0.00	0.00	27.84	5.29	0.00	0.00
part	84.13	13.01	0.61	0.00	0.00	22.31	0.37	0.00	0.00
dec + part	85.54∗	9.91	4.03	0.00	0.00	21.41	1.73	0.00	0.00
dverge	80.54	39.58	17.82	0.10	0.00	36.70	14.58	0.00	0.00
adv	70.98	53.38	33.55	0.62	0.37	53.38∗	33.55	0.62	0.37
dec + adv	65.68	53.95∗	40.41∗	11.23∗	7.74∗	53.08	39.34∗	5.32	2.07∗
dec + part + adv	65.60	40.50	26.26	9.78	6.16	40.49	20.50	7.49∗	1.67

$R_{iu}$**(% AUC)**									

baseline	30.01	27.27	27.13	44.65	48.54	26.58	31.31	56.10	59.36
dec	28.02	28.35	29.79	38.90	39.90	28.59	34.87	41.94	44.29
part	31.47	57.56	54.23	50.79	50.95	52.42	56.14	53.37	54.94
dec + part	35.65	63.60∗	65.06	76.09∗	76.42∗	60.90	69.17∗	76.09∗	77.32∗
dverge	16.77	16.83	18.26	26.12	27.94	16.80	19.97	31.56	35.93
adv	59.11∗	62.30	66.15∗	74.85	75.02	62.30∗	66.15	74.85	75.02
dec + adv	29.77	29.94	28.67	28.25	29.31	29.52	28.23	32.83	37.35
dec + part + adv	40.52	49.41	52.39	61.73	61.34	47.73	52.10	61.07	60.51

UA**(% AUC)**									

baseline	77.91∗	44.22	32.81	44.65	48.54	41.72	33.00	56.10	59.36
dec	75.82	43.58	34.38	38.90	39.90	41.94	36.36	41.94	44.29
part	70.20	52.82	53.61	50.79	50.95	52.49	55.80	53.37	54.94
dec + part	71.54	55.16∗	61.77∗	76.09∗	76.42∗	56.01∗	66.39∗	76.09∗	77.32∗
dverge	75.85	43.90	29.97	26.16	27.94	42.10	29.06	31.56	35.93
adv	51.38	53.67	59.98	74.24	74.52	53.67	59.98	74.24	74.52
dec + adv	60.84	54.55	46.81	33.63	32.81	53.92	46.27	34.27	37.62
dec + part + adv	54.97	50.56	51.60	59.29	59.77	50.06	50.63	59.41	59.96

$R_{cc}$, correct-certain ratio; $R_{iu}$, incorrect-uncertain ratio; UA, uncertainty accuracy. Asterisks indicate the best score for a metric.

### Uncertainty difference between incorrect and correct predictions

Since uncertainty $I_{norm}$ should be a useful discriminative feature for distinguishing correct and incorrect predictions, it is desirable that incorrect predictions, on average, output higher uncertainty than correct predictions. Thus, we simply define the average difference in uncertainty between incorrectly and correctly classified samples:

$$\Delta I_{norm}=E\left[I_{norm}|incorrect\right]-E\left[I_{norm}|correct\right]\text{.}$$

**Figure 2:**
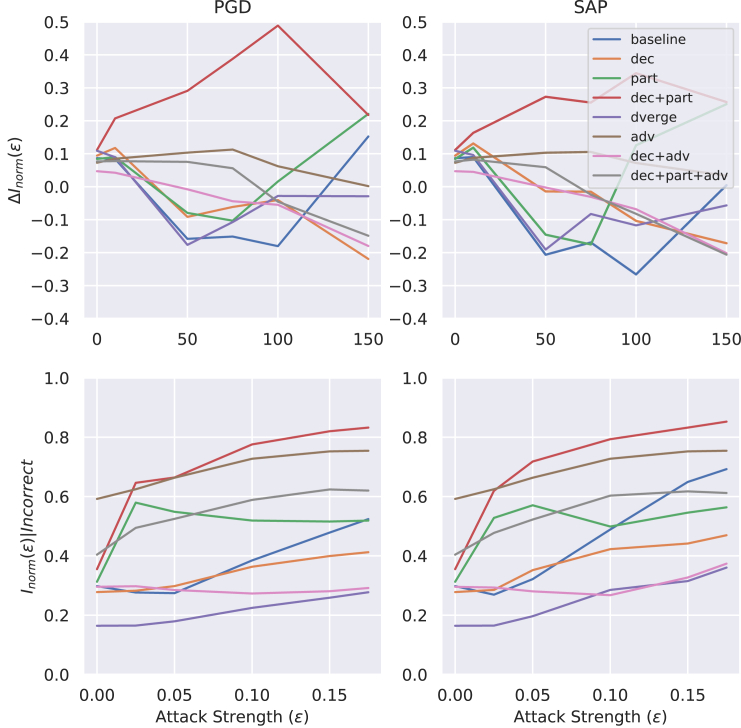
Uncertainty differences from various adversarial attacks Top: PhysioNet 2017 difference in average normalized uncertainty between incorrect and correct samples with varying attacks. Bottom: CPSC 2018 average uncertainty on incorrectly classified samples with varying attacks. The x axis for all plots is the magnitude ε of PGD (left) or SAP (right) adversarial attack.

Figure 2 compares this $\Delta I_{norm}$ for all PhysioNet experiments and ensembles as a function of attack strength ε. For CPSC 2018 experiments, the average uncertainty for incorrect samples is observed, since ensemble accuracy at higher attack magnitudes is too low to estimate $E\left[I_{norm}|correct\right]$. Higher values are desirable for both measures. While comparisons between different ensembles are largely heterogeneous, the dec + part most consistently scores the highest across different attack conditions, suggesting better detection of adversarial samples via uncertainty estimation. adv also outperforms other methods (excluding dec + part) in the majority of cases with regard to these measures.

### Performance on a partially attacked dataset

In a clinical setting, deep models should be used to augment clinical workflows. When the amount of data needing analysis outstrips the available time of qualified clinicians, deep ensembles can initially assess all inputs and defer the most uncertain samples to human readers. This begs the question, “If a deep ensemble is budgeted a certain number of cases that it can refer to human experts, then how many cases would still be misclassified?” To investigate this, we ran the following hypothetical experiment: (1) a dataset of all natural samples and a partially perturbed dataset were drawn. In the partially perturbed dataset, only 50% of the data were unperturbed, and 25%, 15%, and 10% of the data had ε=10,50,75 and ε=0.025,0.05,0.15 perturbations for the PhysioNet and CPSC data, respectively. (2) Each ensemble evaluated all samples, ordering the inferences from most to least confident. (3) Starting with the most confident samples, varying amounts of samples would defer to the deep model’s classification, while all other (less confident) samples would be correctly classified, presumably reviewed by clinicians. Note that this experiment is not meant to exactly reflect the actions and metrics of a true clinical workflow; rather, it is to investigate and compare the potential benefit of the aforementioned deep ensemble methods in augmenting human workers, particularly in the face of adversarially corrupted data.

**Figure 3:**
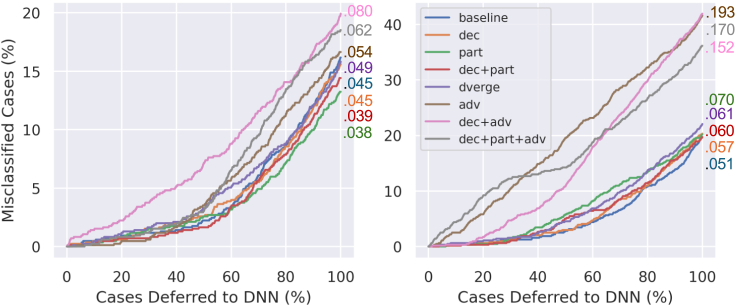
Misclassification and deferral analysis on a natural dataset Percentage of misclassified cases with respect to percentage of cases deferred to the deep ensemble in the natural-only dataset experiments for PhysioNet 2017 (left) and CPSC 2018 (right). Numbers are AUCs (lower is better).

**Figure 4:**
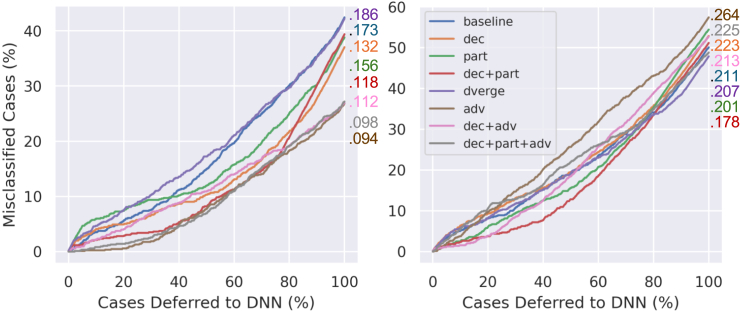
Misclassification and deferral analysis on a partially attacked dataset Percentage of misclassified cases with respect to percentage of cases deferred to the deep ensemble in the partially attacked dataset experiments for PhysioNet 2017 (left) and CPSC 2018 (right). Numbers are AUCs (lower is better).

Figures 3 and 4 plot the percentage of misclassified instances in the sample as a function of the percentage of cases referred to the deep ensemble for the natural and partially perturbed datasets, respectively. In addition, the total AUCs are shown, with lower values being better in this instance. Initially, one can see that adv, dec + adv, and dec + part + adv perform poorly on the natural datasets, particularly the CPSC 2018 data. dec, part, and dec + part perform better than the baseline on the partially perturbed datasets while still performing comparable to or better than the baseline on the natural PhysioNet 2017 dataset. Baseline performs marginally better than dec, dec + part, and dverge in the natural CPSC data, but dec + part outperforms all other methods considerably in the perturbed instance.

## Discussion

Table 1 reflects the accuracy and uncertainty scores of tested ensembles on the PhysioNet 2017 data. Overall, it can be seen that decorrelation and partitioning do not negatively impact natural accuracy in this instance: in fact, overall ensemble accuracy, $R_{cc}$ and $R_{iu}$, are marginally higher on natural data in the dec, part, and dec + part compared to baseline. Furthermore, all ensembles, including the baseline, are somewhat robust to small-magnitude perturbations: Han et al.^39^ reported that their ε=10 SAP attacks fooled a single network 74% of the time, but our results show only a 33.18% error rate for the baseline ensemble under these conditions. This indicates that in this instance, fooling multiple models is more difficult than fooling a single model, even without diversifying measures.^12^ However, this initial robustness plummets in the face of more challenging, larger magnitude attacks, as seen in Table 1. Introducing decorrelation seems to improve robustness in both a regular and an adversarially trained ensemble, with dec + adv achieving the best accuracy on stronger attacks. While partitioning does not benefit classification accuracy, it improves uncertainty estimation, as seen with higher *UA*, with dec + part outperforming even the more expensive adversarially trained ensembles on this metric. These observations all suggest that improved network diversification that via features decorrelation can improve accuracy and uncertainty estimation, while Fourier partitioning improves only uncertainty estimation.

Table 1 also indicates little to no benefit from DVERGE training on the PhysioNet 2017 data; a small trade-off of natural accuracy for adversarial accuracy is seen in the CPSC 2018 data (Table 2). It should be noted that the original intent and testing of DVERGE was on black-box attacks, where the adversary has no direct knowledge of the target model’s parameters.^51^ Thus, it is possible that this method’s effectiveness is reduced when subject to much stronger white-box attacks, as is the case in our experiments, where the adversary can still find points of common instability among all models.

One can see that while adversarial training minimally affects natural performance on the PhysioNet data, natural performance is greatly degraded for adv, dec + adv, and dec + part + adv in the CPSC data in Table 2. This degradation has been observed for adversarial training and is likely due to the network’s limited capacity, which forces a trade-off between natural discriminative features and adversarially robust features.^27^^,^^34^ Indeed, as the dimension of the input space increases, as is the case of the higher-dimensional CPSC data, much larger networks and more training (both adversarial and natural) are needed to fit the robust but complex decision boundaries,^12^ rendering adversarial training less feasible for high-dimensional problems.

Decorrelation alone also provides little to no benefit on the CPSC 2018 experiments; we suspect that this is due to the increase in classes and input dimension. As the number of classes increases, the final feature layer, where decorrelation takes place, must increase in dimension such that feature vectors extracted from different networks can be uncorrelated while still correlating with the correct class. In addition, the expanded input space increases the overlap of unstable points among models, which the decorrelation mechanism must reduce. Despite this, combining dec with other methods such as adv or part yields more robust results compared to any individual method: dec + part generally shows the best *UA*, while dec + adv or dec + part + adv has superior accuracy against perturbed data. Thus, decorrelation still benefits other robustness methods in these higher-dimensional, higher-class count problems. However, as the benefits of using dec + part + adv over dec + adv or dec + part are not consistent across attack strengths in our experiments, benefiting from three methods simultaneously may require more sensitive hyperparameter tuning and more research efforts.

Unlike adversarial training, the combination of dec + part does not substantially change natural or adversarial accuracy, but results in the best UA in all adversarial instances. This suggests that the combination dec + part can improve adversarial uncertainty estimation without sacrificing natural accuracy, even in higher-dimensional problems.

While the metrics in Tables 1 and 2 overall suggest robustness benefits for linear feature decorrelation and Fourier partitioning, these results are admittedly heterogeneous. It is clear that, especially in higher-dimensional data, there is a strong trade-off between natural performance and adversarial robustness. Furthermore, an ensemble’s inference accuracy is not always correlated with the utility of its uncertainty estimation: some ensembles may pose better overall accuracy but worse UA, $R_{iu}$, and/or $R_{cc}$. It is important to evaluate Bayesian neural networks (BNNs) in a method similar to how they could be deployed to observe potential trade-offs between these qualities. This is the motivation behind the mixed dataset experiments (Figures 3 and 4), as it initially explores how well the uncertainty measures of different ensembles can prioritize clinician attention. Figure 3 shows the worst performance on natural data with adversarially trained networks, reflecting the loss of natural performance with adversarial training. On the other hand, dec, part, and dec + part all perform comparably to or better than the baseline and all other methods in the PhysioNet data; dec + part also performs only marginally worse than the baseline in the natural CPSC 2018 data while showing superior performance on both perturbed datasets. Indeed, Figure 4 shows dec, part, and dec + part outperforming both dverge and the baseline of the PhysioNet data in this scenario, and dec + part achieves the best overall performance (smallest AUC) on the perturbed CPSC data. In addition, Figure 2 illustrates that dec + part generally achieves the highest uncertainty difference $\Delta I_{norm}$ on the PhysioNet and the highest average uncertainty on incorrect samples in the CPSC data across varying attack magnitudes.

Our results generally show that the proposed modified linear feature decorrelation and Fourier partitioning methods show promise for diversifying extracted features in deep ensembles and can be used in other high-dimensional classification problems, such as medical image analysis. Specifically, using the fast Fourier transform, Fourier partitioning is an efficient way to force ensemble models to extract different features, which improves uncertainty estimation against white-box ensemble attacks. Previous work mentioned several challenges with scaling decorrelated ensembles to larger problems, such as the need to train models in parallel and the large batch size needed to overdetermine the feature space with each training step.^50^ We find that our modifications, such as selecting the final hidden layer for decorrelation and compressing the feature space with random projections, have allowed us to scale this mechanism to higher dimensions (see details in the methods).

Furthermore, we found that both methods added little to no extra training time and penalized natural accuracy less than adversarial training. It should also be noted that both methods are orthogonal to gradient-based methods such as adversarial training and DVERGE and thus can be combined with these methods. Indeed, our results generally support that using decorrelation can improve the results of adversarial training.

### Limitations of the study

A number of limitations exist for this study, which can be explored in future work:

(1) Optimization of the design space: the introduction of Fourier partitioning and decorrelation introduces new hyperparameters for tuning. For decorrelation, the compression ratio for the features, dimension of the feature space, and batch size are all critical considerations. As previously discussed, we believe that expanding the feature space may be necessary as the number of discriminative classes increases. Fourier partitioning was inspired by the discovery that neural networks can often solve computer vision tasks with only partial frequency information.^34^ Thus, each ensemble filter should be designed to preserve sufficient information for the task but have non-overlapping vulnerabilities. Our experiments simply used two “ring filters,” which summed to an impulse response, but many other schemes could be explored in the same spirit. In addition, these methods can be used in combination with DVERGE or adversarial training. Future experiments should explore all these considerations extensively and introduce new applications as well.
(2) Investigation of robustness against various attacks, corruptions, and shifts: this work focuses on adversarial attacks in ECG of varying magnitude, using both PGD and the more domain-specific SAP algorithms. However, robust uncertainty quantification is desirable in many other contexts, such as domain-specific noise corruption, data domain shifts, and out-of-sample detection. Since neither decorrelation nor Fourier partitioning explicitly optimizes against adversarial attacks, we hypothesize that their diversification benefits may extend to these other contexts.
(3) Experiments mimicking clinical deployment: the trade-offs between a model’s inference accuracy and *UA* make it necessary to test how deep models can work in synergy with clinicians. Our experiments compare the number of misclassified samples when uncertainty is used to theoretically prioritize clinician attention. In reality, however, clinical workflows are more complicated, and desirable outcomes will depend on the cost of false positives/negatives for different diagnoses, clinical resources, and the ability of human clinicians to more accurately diagnose certain diseases relative to a model. Thus, all these aspects should be considered in future translative work. Furthermore, we tested only a natural sample and an attacked sample where the distribution of attack magnitudes was roughly based on the assumption that larger perturbation attacks will be less common. Models should be rigorously tested specifically with the kinds and magnitudes of perturbations that one might expect in deployment.

### Conclusion

Efficient and accurate confidence measurement is necessary for trust in AI systems. We have presented a novel approach for diverse network ensembles using two unique training methods that add little to no training time: a streamlined and accelerated decorrelation training strategy and a Fourier partitioning scheme. These ensembles achieve robustness by focusing on feature diversity between models. In addition, we adapt adversarial training to ensembles and test all methods along with DVERGE in the Bayesian ensemble framework. All approaches are applied to ECG classification with uncertainty estimation and tested for stability against state-of-the-art adversarial ECG attacks, demonstrating their merits and potential in solving large problems. Incorrect diagnoses can cause major harm in many healthcare tasks where AI can work alongside clinicians; predicting model confidence in these contexts is crucial. Thus, we speculate that diverse ensembles will play a key role in elevating trustworthiness and confidence in AI for applications such as tomographic image processing, radiomics, and multimodal diagnosis. We see applications of this approach for robust uncertainty estimation with a diversified ensemble, which discourages different models from extracting redundant features.

## Methods

### Ensemble training and inference

Basic ensembles consist of multiple DNNs trained for the same task. An ensemble inference on sample *x* is simply the average output of each model in the ensemble. Thus, for an ensemble with models $f_{1},f_{2},\ldots f_{K}$:

(Equation 1) $$\hat{y}=f_{ens}\left(x\right)=\frac{1}{K}\sum_{k=1}^{K}f_{k}\left(x\right)\text{.}$$

Note that for a classification task, this output is a discrete probability distribution.

For estimating epistemic uncertainty, we adopt the approach from Mobiny et al.,^54^ which defines the uncertainty of sample *x* as the mutual information between the inferred label $\hat{y}$ and the underlying parameter distribution, in other words, how much additional information sample *x* tells us about the true parameters:

$$I\left(y,\theta |x,D\right)=H\left(y|x,D\right)-H\left(y|x,\theta ,D\right)=H\left(y|x,D\right)-E_{\theta |D}\left[H\left(y|x,\theta \right)\right]\text{.}$$

D is the training data. The first term is intractable, but can be estimated using the network ensemble as the entropy of the expected inference.^54^ Thus, for an ensemble with $f_{1},f_{2},\ldots f_{K}$ models, each of which outputs a discrete probability distribution over *C* classes:

$$I\left(y,\theta |x,D\right)=-\sum_{c=1}^{C}f_{ens}\left(x\right)\left[c\right]\log\;f_{ens}\left(x\right)\left[c\right]+\frac{1}{K}\sum_{k=1}^{K}\sum_{c=1}^{C}f_{k}\left(x\right)\left[c\right]\log\;f_{k}\left(x\right)\left[c\right]\text{.}$$

The scale of *I* is relative and can vary between models and ensembles. Thus, we normalize the uncertainty with the minimum and maximum uncertainty values found during training.

$$I_{norm}=\frac{I-I_{\min}}{I_{\max}-I_{\min}}\text{.}$$

Note that test samples can have greater or less uncertainty than any sample encountered in the training set. Thus, values for $I_{norm}$ are not necessarily limited to [0,1]. For a threshold $I_{T}$, which classifies samples as either “certain” or “uncertain,” metrics $R_{cc}$, $R_{iu}$, and UA were calculated empirically over an adversarial dataset based on the number of correct and certain, correct and uncertain, incorrect and certain, and incorrect and uncertain samples.

For the baseline ensemble, each model was trained independently in sequence. Other ensembles were trained using the methods described below.

### Decorrelation training

#### Decorrelation of two networks

The intent of decorrelation training between two networks is to minimize the Pearson correlation coefficient between the latent features extracted by the two networks, as explained in Wiedeman and Wang.^50^ Minimizing this value incentivizes the networks to extract different features for a task, reducing the transferability of network weaknesses.^27^^,^^50^ Assume two classification models, $f_{1}$ and $f_{2}$, and sample batch *X* of inputs with accompanying labels *Y*. Next, $Z_{i}=f_{i}^{l}\left(X\right)$ for i=1,2 are the latent feature extracted by model *i* at layer *l* from batch *X*. Note that $Z_{i}\in R^{N\times D}$ where *N* is the batch size and *D* is the dimension of the latent space. A linear relationship estimating $Z_{2}$ from $Z_{1}$ with weights *W* can be found using ordinary least-squares regression:

$$\begin{array}{l} \underset{W}{\text{minimize}}\|Z_{2}-Z_{1}W{\|}_{2}^{2}\\ \text{where}\;Z_{1}=\left[Z_{1},1\right]\\ \min_{W}\|Z_{2}-Z_{1}W\left|{|}_{2}^{2}=\|Z_{2}-Z_{1}{\left(Z_{1}^{⊤}Z_{1}\right)}^{-1}Z_{1}^{⊤})Z_{2}\right|{|}_{2}^{2}=SS_{res}\text{.} \end{array}$$

The Pearson correlation coefficient is then the ratio between the residual and the total sum of squares:

$$R^{2}=1-\frac{SS_{res}}{SS_{total}}=1-\frac{\|\left(I-Z_{1}{\left(Z_{1}^{⊤}Z_{1}\right)}^{-1}Z_{1}^{⊤}\right)Z_{2}|{|}_{2}^{2}}{\|Z_{2}-\bar{Z_{2}}|{|}_{2}^{2}}\text{.}$$

To reduce this term during training, the decorrelation loss is defined as:

(Equation 2) $$L_{R}=\log\left(SS_{total}+\epsilon \right)-\log\left(SS_{res}+\epsilon \right)\text{,}$$

(Equation 3) $$L_{R}\left(Z_{1},Z_{2}\right)=\log\left(\|Z_{2}-\bar{Z_{2}}|{|}_{2}^{2}+\epsilon \right)-\log\|\left(I-Z_{1}{\left(Z_{1}^{⊤}Z_{1}\right)}^{-1}Z_{1}^{⊤}\right)Z_{2}|{|}_{2}^{2}+\epsilon )\text{,}$$

where ϵ is some small constant for stability (set to $10^{-5}$ in our experiments) and $\bar{Z_{2}}$ is an N×D matrix where each row is the sample mean of $Z_{2}$. This decorrelation can be applied to model training simply by weighting and adding this loss to a conventional training objective (e.g., cross-entropy loss) for both networks, balancing feature decorrelation and individual network performance.

#### Scaling decorrelation to ensembles

While decorrelation of two networks was shown to reduce transferability of adversarial attacks in Wiedeman and Wang,^50^ several issues impede its use in larger ensembles and higher-dimensional problems. First, this method requires training multiple networks in parallel to obtain $Z_{1}$ and $Z_{2}$, which linearly scales the memory needed for training and quickly becomes infeasible for more networks, larger networks, and larger data sizes. Second, calculating the loss in Equation 3 requires taking the pseudo-inverse of $Z_{1}\in R^{N\times D+1}$, requiring batch size N>D+1 for a sufficiently overdetermined system. Higher-dimensional problems often necessitate higher-dimensional latent spaces and smaller batch sizes, rendering this condition impractical.

To solve the above problems and extend the method to ensembles consisting of more than two networks, we employ the following modifications:

(1) Selecting the regression layer: most deep classification networks can be divided into a multi-layer feature extractor and a linear layer followed by a softmax function (i.e., a logistic regression). We select the network layer just before this final linear layer, as it represents the highest-level features and is typically lower dimension than previous layers.
(2) Dimensionality reduction via random projections: prior to computing the pseudo-inverse of the regressor, we compress its D+1 dimensionality to r<D+1 by applying a random projection, $R\in R^{D+1\times r}$. To balance the asymmetry in this relationship, we randomly select which network’s extracted features act as the regressor and which as the regressand with each training batch. Our new loss is expressed as follows:

$$\begin{array}{l} L_{R}^{*}\left(Z_{1},Z_{2}\right)=\left\{ \begin{array}{cc} L_{R}\left(Z_{1},Z_{2}R\right) & \text{with}\;\text{prob}.\;0.5\\ L_{R}\left(Z_{2},Z_{1}R\right) & \text{with}\;\text{prob}.\;0.5 \end{array}\right.\\ R\in R^{D+1\times r}∼N\left(0,1/\sqrt{D}\right)\text{.} \end{array}$$

Although these projections individually do not capture all information in the regressor, they are drawn randomly with each training batch, preventing the networks from decorrelating only a subspace of the original feature space.

(1) Models are trained in sequence instead of parallel: the first network is trained without any decorrelation loss. After a model is trained, its extracted features on all training samples are saved. While the next model is being trained, these features are loaded with the corresponding batch samples and then used for decorrelation. As such, rather than dynamically decorrelating multiple networks at once, which requires simultaneous training of all networks, we simply use the features extracted by the previously trained networks as constant values to decorrelate against. For decorrelating against multiple models, we average the modified correlation loss against all the previously trained models. Thus, the entire decorrelation loss for model *k* in an ensemble is:

(Equation 4) $$L_{cor}\left(Z_{k},Z_{k-1}\cdots Z_{0}\right)=\frac{1}{k}\sum_{i=0}^{k-1}L_{R}^{*}\left(Z_{k},Z_{i}\right)\text{.}$$

#### Final decorrelation scheme

**Figure 5:**
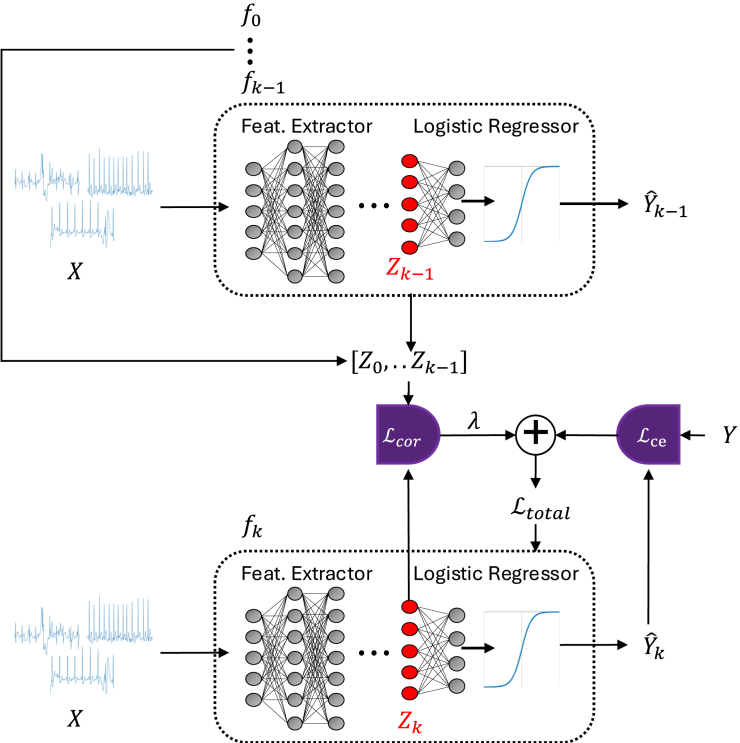
Decorrelation training process illustration The current model $f_{k}$ is trained end to end (feature extractor and regressor) using both cross-entropy and our correlation loss. The correlation loss references previous models’ extracted sample features as opposed to training multiple models in parallel.

Figure 5 illustrates the sequential training of the decorrelated ensemble. The total loss for model *k* on batch $\left(X_{b},Y_{b}\right)$ with extracted features from $X_{b}$ using k−1 previous models as $\left(Z_{k-1,b},\ldots Z_{0,b}\right)$ is:

(Equation 5) $$L_{total}=L_{ce}\left(f_{k}\left(X_{b}\right),Y_{b}\right)+\lambda L_{cor}\left(Z_{k,b}Z_{k-1,b}\cdots Z_{0,b}\right)\text{,}$$

where $L_{ce}$ is the cross-entropy loss and λ is a weighting hyperparameter. The implementation is summarized in the Algorithm 1. While stochastic gradient descent is shown here, any optimizer can be used for the gradient step.

Algorithm 1 **Training step for model fk (with parameters θk) using decorrelation. λ and r are hyperparameters.**

Draw $X_{b},Y_{b},\left[Z_{k-1,b}\cdots Z_{0,b}\right]$ ▷ Draw training batch and corresponding features from prior models

$\begin{array}{l} Z_{k,b}\leftarrow f_{k}^{l}\left(X_{b}\right)\\ N,D\leftarrow shape\left(Z_{k,b}\right)\\ \hat{Y_{b}}\leftarrow f_{k}\left(X_{b}\right)\\ L\leftarrow L_{ce}\left(\hat{Y_{b}},Y_{b}\right)\\ i\leftarrow 0 \end{array}$

**while** i≤k−1 **do**

 
$Z_{1},Z_{2}\leftarrow Z_{k,b},Z_{i,b}$

 
**if** t∼Uniform[0,1]<0.5 **then** $Z_{1},Z_{2}\leftarrow Z_{2},Z_{1}$

 
**end if**

 
$R∼N\left(0,1/\sqrt{D}\right)\in R^{D+1\times r}$

 
$Z_{1}\leftarrow \left[Z_{1},1\right]R$

 $L\leftarrow L+\frac{\lambda }{k}L_{R}\left(Z_{1},Z_{2}\right)$ ▷ Apply decorrelation loss from Equation 3

 
i←i+1

**end while**

$\theta_{k}\leftarrow \theta_{k}-\eta \nabla_{L}\theta_{k}$

### Fourier partitioning scheme

**Figure 6:**
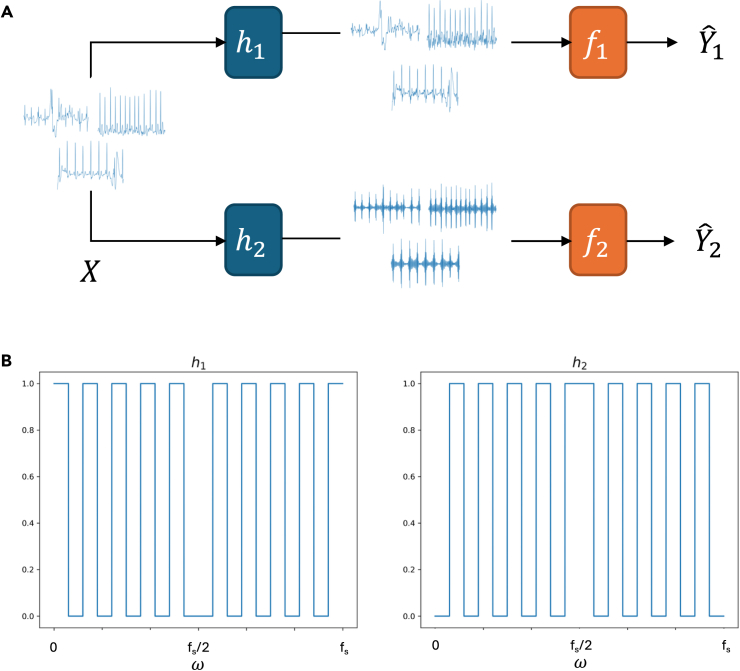
Fourier transform-based input data decomposition (A) Diagram of frequency partitioning where each sample is split into two inputs fed to separate models, with *h* as a partitioning filter and *f* as a classification model. (B) Frequency responses of $h_{1}$ and $h_{2}$ filters, with $f_{s}$ as the data sampling frequency (300 Hz for PhysioNet 2017 and 500 Hz for CPSC 2018).

With the Fourier partitioning scheme, the first ensemble model has no modification. The other models were trained normally, but inputs were filtered during both training and inference (Figure 6). This approach is inspired by Yin et al.,^34^ who showed that neural networks can achieve high classification accuracy in many computer vision tasks with only a portion of an input’s frequency data and that models often overfit to discriminative features in specific frequency bands:

$${\hat{y}}_{i,k}=f_{k}\left(h_{k}⊛x_{i}\right)\text{.}$$

In practice, filter convolution was done by point-wise multiplication in the Fourier domain, computed using the fast Fourier transform.

### Adversarial attacks

Adversarial attacks are formulated by maximizing the loss objective *L* of the model *f* by modifying *x* (with the paired label *y*) within a set of valid perturbations Δ (i,e., perturbations that do not change the underlying semantic content of the input):

(Equation 6) $$\underset{\delta }{\text{maximize}}\;J\left(x+\delta ,y\right)$$

subjecttoδ∈Δ(x).

Two algorithms were used to craft adversarial attacks: PGD and SAP. PGD is widely used as a strong, iteratively optimized attack, where Δ is defined as a small, $l_{\infty }$ ball^12^:

(Equation 7) $$x_{i}^{\prime }={\text{Clip}}_{\varepsilon }\left(x_{i-1}^{\prime }+\alpha \text{sgn}\left(\nabla_{x}L\left(f\left(x_{i-1}^{\prime }\right),y\right)\right)\right)\text{.}$$

$x_{i}^{\prime }$ is the sample at the $i^{th}$ iteration, *y* is the corresponding label, α is a step size, and the clipping operation clips all values to be within the $l_{\infty }$ ball of radius ε around *x*, as well as any implicit bounds on the domain of *X*.

The attack algorithm targets a full ensemble, $f=f_{ens}$, by applying the cross-entropy loss as *L* to ensemble inference from Equation 1, which yields:

$$L\left(f_{ens}\left(x_{i-1}^{\prime }\right),y\right))=-\log\frac{1}{K}\sum_{k=1}^{K}f_{k}\left(x_{i-1}^{\prime }\right)\left[y\right]\text{.}$$

We found this approach to be impractical, since the gradient must be backpropagated through all ensemble models simultaneously with each iteration. However, since −log is convex and the ensemble inference is an average of multiple points, Jensen’s inequality produces an upper bound on this loss, which we optimize as a surrogate^55^:

$$\begin{array}{l} -\log\frac{1}{K}\sum_{k=1}^{K}f_{k}\left(x_{i-1}^{\prime }\right)\left[y\right]\leq \frac{1}{K}\sum_{k=1}^{K}-\log\;f_{k}\left(x_{i-1}^{\prime }\right)\left[y\right]\\ \leq \frac{1}{K}\sum_{k=1}^{K}L\left(f_{k}\left(x_{i-1}^{\prime }\right),y\right)\text{.} \end{array}$$

Thus, computing the gradient $\nabla_{x}$ of this upper bound is equivalent to taking the gradient with respect to each individual model loss and averaging these results. Rather than compute forward and backward passes for all *K* models at each iteration, we find it effective to simply alternate the target model with each iteration:

(Equation 8) $$\begin{array}{l} x_{i}^{\prime }={\text{Clip}}_{\varepsilon }\left(x_{i-1}^{\prime }+\alpha \text{sgn}\left(\nabla_{x}L\left(f_{k}\left(x_{i-1}^{\prime }\right),y\right)\right)\right)\\ \text{where}\;k=i\%K\text{.} \end{array}$$

SAP is a variation of PGD designed to craft smooth attacks for ECG signals. The details of SAP can be found in Han et al.^39^ To summarize, perturbation θ is iteratively optimized in a fashion similar to PGD, but it is also convolved with a sequence of *M* Gaussian kernels at every step, each of which is parameterized by its width s and standard deviation σ:

(Equation 9) $$\begin{array}{l} \theta_{i}={\text{Clip}}_{\varepsilon }\left(\theta_{i-1}^{\prime }+\alpha \text{sgn}\left(\nabla_{\theta }L\left(f_{k}\left(x^{\prime }\left(\theta_{i-1}\right)\right),y\right)\right)\right)\\ x^{\prime }\left(\theta \right)=x+\frac{1}{M}\sum_{m=1}^{M}\theta ⊛K\left(s_{m},\sigma_{m}\right)\text{.} \end{array}$$

As with the above PGD modification, the target model is alternated with each iteration to craft an ensemble attack. The convolution with Gaussian kernels smooths high-frequency perturbations, removing unrealistic square-wave artifacts. PGD and SAP attacks were optimized over 20 steps in total. For both attacks, α was scaled as ε/10. All adversarial attacks were crafted from the validation to target one of the models in an ensemble. For experiments with PhysioNet 2017 data, the convolution kernels are identical to those used in Han et al.^39^: s=(5,7,11,15,19),σ=(1,3,5,7,10). For the CPSC 2018 experiments, we used s=(9,11,15,19,21),σ=(5,7,10,13,17).

### Experiment details

The two ECG datasets used in this work are from the PhysioNet 2017 and CPSC 2018 challenges. The PhysioNet dataset contains 30- to 60-s-duration single-channel ECG recordings sampled at 300 Hz. All samples were zero-padded to be 60 s. Class labels were normal, A. Fib., other rhythm, and noise. Scaling of the signal magnitudes was identical to those in Han et al.^39^ The CPSC 2018 data contained 6- to 60-s ECG recordings at 500 Hz with nine class labels: normal, A. Fib, 1^st^-degree atrioventricular block, left bundle branch block, right bundle branch block, premature atrial contractions, premature ventricular contraction, ST-segment depression, and ST-segment elevated. All samples were truncated or zero-padded to be 48 s. For simplicity, the minority of samples labeled with multiple diagnoses were not used. In addition, all channels were normalized from −1 to 1.

For all experiments, each ensemble consisted of K=3 classification networks, each with the architecture used in Goodfellow et al.^6^ for experiments using the PhysioNet 2017 data. For experiments using the CPSC 2018 data, this architecture was simply modified to have 12 input channels and 9 output classes. Each network was trained for 80 epochs (batch size of 64) using the Adam optimizer with a learning rate of $10^{-3}$. Pytorch 1.8.1 was used with two NVIDIA Titan RTX GPUs and a 90/10 training/validation split. For all ensembles using decorrelation training, hyperparameters r=32 and λ=0.2 were used (the uncompressed latent dimension *D* was 64).

For ensembles that underwent additional ensemble adversarial training, each model in the ensemble was sequentially trained using adversarial samples. In practice, this is identical to regular training, except each sample batch is perturbed using PGD (Equation 7) prior to the forward training step. The perturbations target the model being trained and use ε=10 and 0.025 for PhysioNet 2017 and CPSC 2018, respectively. Each model was trained for an additional 2 h, translating to 6 extra training hours total for each adversarially trained ensemble.

DVERGE training is similar to adversarial ensemble training in that both use PGD to perturb a sample $x_{s}$ within a small $l_{\infty }$ bound. Differences are that in DVERGE, (1) this optimization is used to maximize similarity between the distilled features of $x_{s}$ and some other randomly drawn sample *x* at some randomly selected feature layer *l* of the network and (2) the samples perturbed using network *i* are used to train *other* networks i≠j. Thus, we use the feature distillation objective and training procedure from Yang et al.^51^:

(Equation 10) $$\begin{array}{l} x_{f_{i}^{l}}^{\prime }\left(x,x_{s}\right)=\underset{z}{\mathrm{argmin}}\|f_{i}^{l}\left(z\right)-f_{i}^{l}\left(x\right)|{|}_{2}^{2}\text{,}\\ \text{subject}\;\text{to}\;\varepsilon \geq {\left\|z-x_{s}\right\|}_{\infty }\text{.} \end{array}$$

We found that deploying this original implementation decreased natural accuracy on PhysioNet 2017 data, where there is a low number (4) of discrete classes. DVERGE creates new samples by distilling the non-robust features of one random sample onto another.^51^ However, if these two samples belong to the same class (which is more likely to occur when there are few classes or class imbalances), the features distilled from one sample may already be similar to the other, negating any benefit. Consequently, we added the following constraint: $\left(x,x_{s}\right)$ batches are drawn such that $y\neq y_{s}$.

Values for ε were identical to those used in adversarial ensemble training. The DVERGE training also ran for the same total training time (6 additional hours) as adversarial ensemble training. For each batch, feature layer *l* was randomly, uniformly selected from the post batch-norm layers of the networks.

## Resource availability

### Lead contact

Requests for further information and resources should be directed to and will be fulfilled by the lead contact, Ge Wang (wangg6@rpi.edu).

### Materials availability

This study did not generate new materials.

### Data and code availability

Data used in this paper are from the 2017 PhysioNet Cardiology Challenge^4^ and the 2018 CPSC.^5^ All original code has been archived at Zenodo at https://doi.org/10.56789/.14052936 and is publicly available as of the date of publication.^56^ An active repository containing our source code is also on GitHub: https://github.com/WANG-AXIS/DNA_ECG.

## Acknowledgments

This work was partially supported by US 10.13039/100000002National Institutes of Health (NIH) grants R01EB026646, R01CA233888, R01CA237267, R01HL151561, R21CA264772, and R01EB031102 and a National Science Foundation Graduate Research Fellowship supporting C.W. The authors thank all members of the lab for their support.

## Author contributions

C.W. and G.W. jointly conceived the idea for this study. C.W. designed code for executing all experiments and drafted the paper. G.W. was heavily involved in supervising the project, interpreting results, and editing the paper.

## Declaration of interests

The authors declare no competing interests.
